# Radio-frequency optomechanical characterization of a silicon nitride drum

**DOI:** 10.1038/s41598-020-58554-x

**Published:** 2020-02-03

**Authors:** A. N. Pearson, K. E. Khosla, M. Mergenthaler, G. A. D. Briggs, E. A. Laird, N. Ares

**Affiliations:** 1 Department of Materials, University of Oxford, Parks Road, Oxford, OX1 3PH United Kingdom; 2 Center for Engineered Quantum Systems, The School of Mathematics and Physics, The University of Queensland, St. Lucia, Queensland 4072 Australia; 3 QOLS, Blackett Laboratory, Imperial College London, London, SW7 2AZ United Kingdom; 4 Department of Physics, Lancaster University, Lancaster, LA1 4YB United Kingdom

**Keywords:** Applied physics, NEMS

## Abstract

On-chip actuation and readout of mechanical motion is key to characterize mechanical resonators and exploit them for new applications. We capacitively couple a silicon nitride membrane to an off resonant radio-frequency cavity formed by a lumped element circuit. Despite a low cavity quality factor (*Q*_*E*_ ≈ 7.4) and off resonant, room temperature operation, we are able to parametrize several mechanical modes and estimate their optomechanical coupling strengths. This enables real-time measurements of the membrane’s driven motion and fast characterization without requiring a superconducting cavity, thereby eliminating the need for cryogenic cooling. Finally, we observe optomechanically induced transparency and absorption, crucial for a number of applications including sensitive metrology, ground state cooling of mechanical motion and slowing of light.

## Introduction

Cavity optomechanics boasts a number of tools for investigating the interaction between radiation and mechanical motion and enables the characterization and development of highly sensitive devices^1^. Silicon nitride membranes have been fabricated to exhibit very high tensile stress, resulting in high quality factors, and have been used for a number of applications including measurement of radiation pressure shot noise^2^, optical squeezing^3^, bidirectional conversion between microwave and optical light^4^, optical detection of radio waves^5,6^, microkelvin cooling^7^ and cooling to the quantum ground state of motion^8–10^.

Radio-frequency (rf) cavities allow for sensitive mechanical readout on-chip^5,11–14^. We characterize a silicon nitride membrane at room temperature making use of an off-resonant rf cavity^13,14^. In our approach, the use of lumped elements greatly simplifies the detection circuit in terms of fabrication and allows the integration on chip with the mechanical oscillator. Our circuit has a lower operation frequency than microwave cavities^11,14^, and allows for a larger readout bandwidth than previous works^13^. Also, our cavity allow us to inject noise, effectively increasing the mechanical mode temperature. We are able to detect several modes and extract the quality factor and cavity coupling strength for each of them. When the membrane is driven, we are able to resolve the membrane’s motion in real time. We achieve a sensitivity of $0.4\,{\rm{pm}}/\sqrt{{\rm{Hz}}}$. A sensitivity of $4.4\,{\rm{pm}}/\sqrt{{\rm{Hz}}}$ was reported in ref. ^14^, although it must be noted that these sensitivities cannot be easily compared, due to the much smaller size of their mechanical resonator. We observe optomechanically induced transparency (OMIT) and optomechanically induced absorption (OMIA) on-chip and deep in the unresolved sideband regime, allowing for the characterisation of the membrane’s motion under radiation pressure. OMIT and OMIA are an unambiguous signature of the optomechanical interaction^15^ and they can be used to slow or advance light^16^. OMIT has also been proposed as a means to achieve ground state cooling of mechanical motion in the unresolved sideband regime^17,18^.

## Experiment

**Figure 1:**
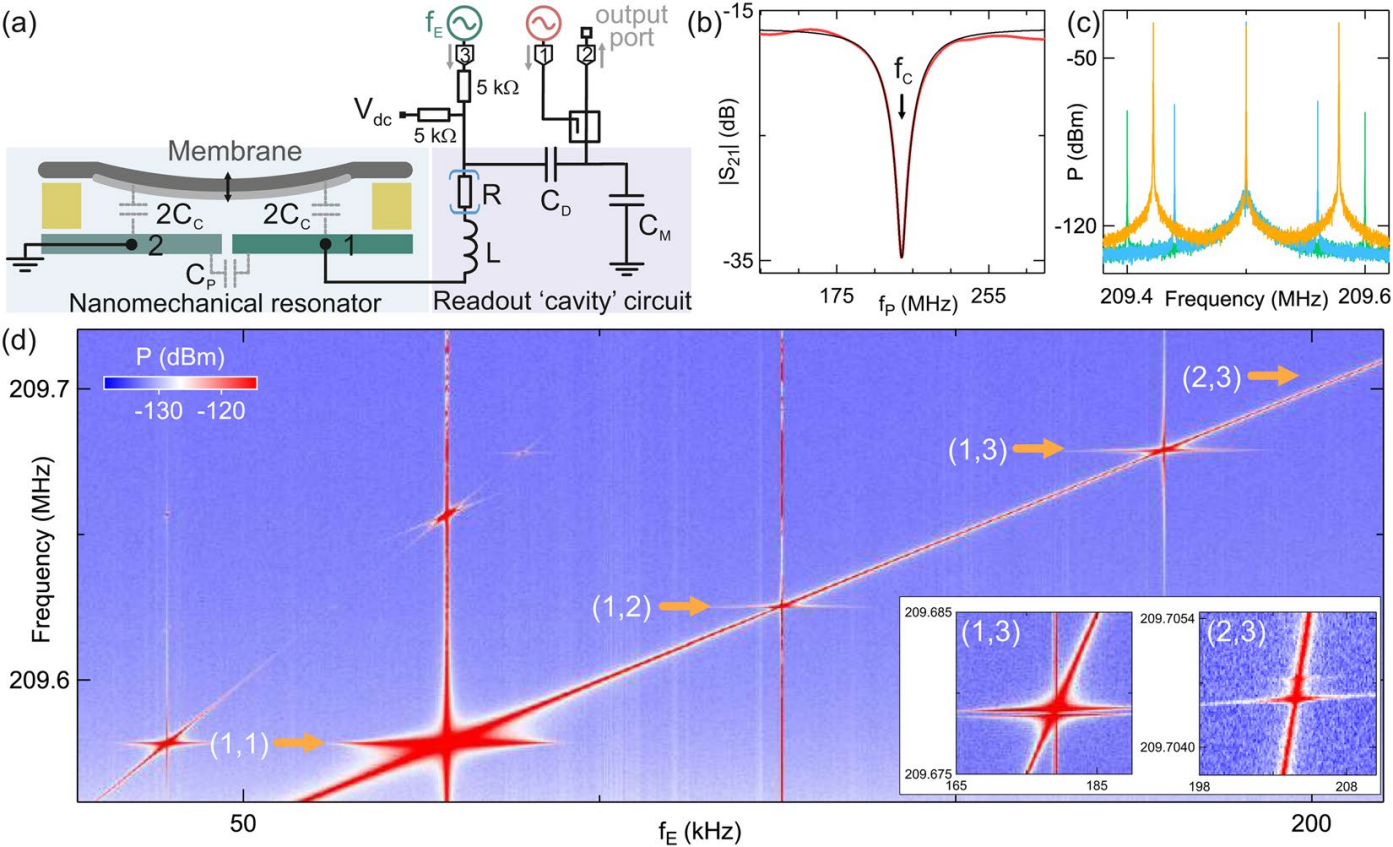
(**a**) Experimental setup. A metalized silicon nitride membrane is suspended over two metal electrodes. Electrode 2 is grounded and electrode 1 is connected to a radio-frequency tank circuit acting as a readout cavity. The cavity is formed from an inductor *L* = 223 nH and 10 pF fixed capacitors *C*_D_ and *C*_M_. Parasitic capacitances contribute to *C*_M_. The role of *C*_M_ is to improve on the impedance matching between the circuit and the 50 Ohm line. *C*_D_ controls the coupling to the cavity, i.e. the number of photons entering the cavity. Because these capacitors are larger than *C*_T_, they do not strongly affect the cavity frequency. Parasitic losses in the circuit are parameterized by an effective resistance *R*. To probe the cavity a radio-frequency signal is injected at port 1, passed via a directional coupler, and after reflection received at port 2. The signal is measured using a vector network analyser or spectrum analyser. The membrane’s motion is excited via port 3. Bias resistors allow a dc voltage *V*_dc_ to be added to the membrane drive signal. (**b**) Reflection as a function of frequency. The black curve is a fit to a circuit model. (**c**) Power spectrum of the signal at port 2 showing the carrier peak at the cavity resonance frequency, applied via port 1, and sidebands corresponding to a frequency modulated tone applied via port 3. The light blue and light green curves correspond to the power spectrum for an excitation *f*_E_ below (60 kHz) and above (100 kHz) *f*_0_ respectively. Sidebands appear due to non-linearities in the circuit. The orange curve corresponds to *f*_E_ ~ *f*_0_. In this case, the sidebands are larger. (**d**) Higher frequency sidebands in (**c**) as a function of *f*_E_. When *f*_E_ is close to a mechanical mode of the membrane, a mechanical sideband appears. The four visible mechanical modes are indicated with arrows. Because higher modes produce fainter features, they are not displayed in this graph. Note that the colour scale displays a range of only 25 dBm in order to reveal the weaker features. Insets: zoom-in of the two highest frequency modes displayed. We observe a splitting of the mechanical feature, indicating nearly degenerate modes.

Our device consists of a high-stress silicon nitride membrane which is 50 nm thick; it has an area of 1.5 mm × 1.5 mm and 90% of this area is metalized with 20 nm of Al. We suspend this membrane over two Cr/Au electrodes patterned on a silicon chip. A dc voltage V_dc_ = 15 V is applied to electrode 1, with electrode 2 grounded. Measurements were performed at room temperature and at approximately 10^−6^ mbar. For optomechanical readout and control, electrode 1 is connected to an effective rf cavity. The cavity is realised using an on-chip inductor *L* and capacitors *C*_D_ and *C*_M_ mounted on the sample holder^19^, in addition to the capacitance formed by the membrane *C*_C_. The circuit behaves similarly to a simple LC resonator with total capacitance *C*_T_ = *C*_C_ + *C*_P_, where *C*_P_ accounts for the capacitance between the two sides of the antenna and other parasitic capacitances. This circuit acts as a cavity and can be driven by injecting an rf signal to the input (port 1) via a directional coupler in order to induce an optomechanical interaction between the cavity signal and the mechanical motion. In addition port 3 allows injection of an ac signal to directly excite the membrane’s motion. The entire setup forms a three-terminal circuit with input ports 1 and 3 and output port 2 (Fig. 1(a)). We used a vector network analyzer to measure scattering parameters and a spectrum analyzer to measure power spectra. Figure 1(b) shows the scattering parameter (|S_21_|) as a function of cavity probe frequency *f*_P_. The cavity resonance is evident in reflection as a minimum in |S_21_| with quality factor *Q*_E_ ≈ 7.4.

The dependence of the capacitor formed between the electrodes and the metalized membrane *C*_C_ on the mechanical displacement *u* leads to coupling between the cavity and the mechanical motion. This coupling is given by $\frac{d{f}_{{\rm{C}}}}{du}\approx 1/(4\pi \sqrt{{L}{{C}}_{{\rm{T}}}^{3}})\frac{\partial {C}_{{\rm{C}}}}{\partial u},$ where *f*_C_ is the cavity resonance frequency, *C*_T_ the total circuit capacitance and *u* the membrane’s displacement from its equilibrium position. The coupling causes phase shifts of the cavity reflection, allowing us to monitor the membrane’s motion in the unresolved sideband limit. The single-photon coupling strength, which measures the interaction between a single photon and a single phonon, is therefore,

1 $$\frac{{g}_{0}}{2\pi }\approx \frac{1}{4\pi \sqrt{{L}{{C}}_{{\rm{T}}}^{3}}}\frac{\partial {C}_{{\rm{C}}}}{\partial u}{u}_{{\rm{ZP}}},$$

where *u*_ZP_ is the amplitude of the membrane’s zero-point motion.

Using a simple circuit model (See Supplemental Material for details of the circuit simulation), we fit |S_21_| and extract *C*_C_ ≈ 1.6 pF. Within the parallel plate capacitor approximation, ${C}_{{\rm{C}}} \sim \frac{{\varepsilon }_{0}a}{d},$ where *ε*_0_ is the vacuum permittivity, *a* is the metallised area of the membrane and *d* is the membrane-electrode gap. From this expression, we extract *d* ~ 9 *μ*m.

## Results

To find the mechanical resonances, we drove the cavity on resonance (*f*_C_ ≈ 209.2 MHz) with input power *P*_C_ = 5 dBm at port 1. Meanwhile, we excited the membrane’s motion via port 3, using a sinusoidal signal at frequency *f*_E_ and with amplitude *V*_M_ = 3.6 V_rms_ at electrode 1. In order for the mechanical response to appear broader in the frequency spectrum, facilitating the detection of the mechanical modes, the excitation frequency at port 3 was modulated with a white noise pattern with a deviation of 200 Hz. The power spectrum *P* of the reflected signal at port 2 shows sidebands at *f*_C_ ± *f*_E_ due to non-linearities of the rf circuit giving rise to frequency mixing. The mechanical signal appears when *f*_E_ is close to a mechanical resonance, and is evident as a pronounced increase in sideband amplitude and width (Fig. 1(c)).

Figure 1(d) shows the sideband at *f*_C_ ± *f*_E_ as a function of *f*_E_. The fundamental mode frequency *f*_1,1_, which we will call *f*_0_, is ~77.9 kHz, giving an unresolved sideband ratio of 2*πf*_0_/*κ* ~ 3 × 10^−3^, with *κ* = 2*πf*_C_/*Q*_E_ the cavity linewidth. As well as the fundamental mode, we observe less strong harmonics *f*_*i*,*j*_ near the expected frequencies. The expected frequencies for higher harmonics theoretically satisfy the ratios ${f}_{i,j}/{f}_{0}=\frac{1}{\sqrt{2}}\sqrt{{i}^{2}+{j}^{2}}$ for integers *i* and *j* with symmetric roles as expected for a square membrane. Two of the sidebands are double peaked, evidencing nearly degenerate mechanical modes (insets Fig. 1(d)). The broken degeneracy could be due to imperfections in how the membrane was fabricated and fixed in place or uneven binding/deposition of the Al layer.

**Figure 2:**
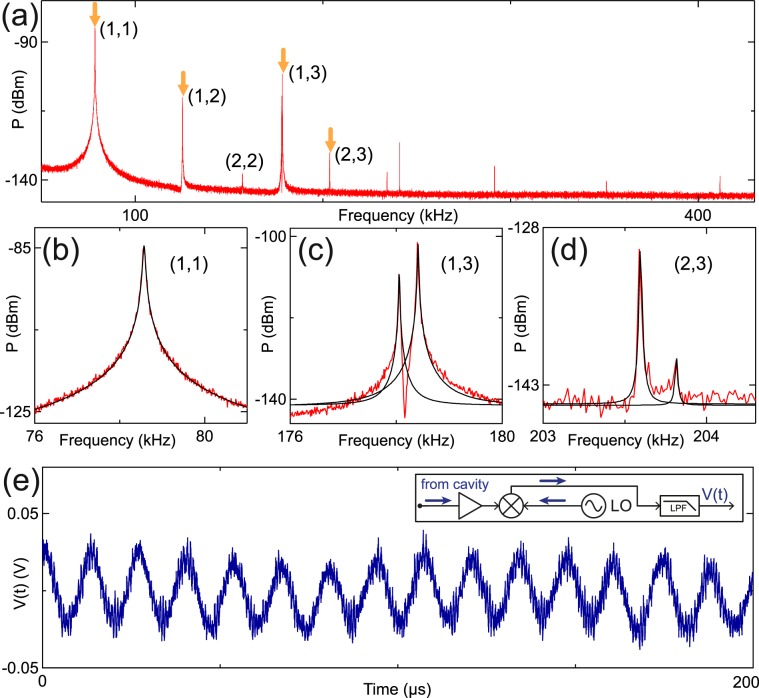
(**a**) Power spectrum as a function of frequency for the higher-frequency sidebands. A tone at *f*_C_ is applied with input power *P*_C_ = 5 dBm and white noise is injected via port 3 at *V*_M_ = 2.7 V_rms_ at electrode 1. We observe several mechanical modes. The four mechanical modes visible in Fig. 1(d) are indicated with arrows. The mode at ~157 kHz, labelled (2,2), is not visible in Fig. 1(d) due to a higher noise floor than in (**a**), which arises from a higher resolution bandwidth. Higher modes are not labelled because the deviation from theoretical ratios makes their identification ambiguous. (**b**–**d**) Zoom in showing the fundamental mode, and selected nearly degenerate modes. The black curves are Lorentzian fits. (**e**) Demodulated output signal as a function of time for the fundamental mode. The demodulation circuit is shown in the inset. (LO: local oscillator; LPF: low-pass filter). Input power is *P*_C_ = 12 dBm at *f*_C _and the white noise is *V*_M_ = 3.6 V_rms_ at electrode 1.

The entire set of mechanical resonances can be observed in a single measured power spectrum by driving the cavity at *f*_C_ and injecting white noise via port 3. White noise excites the motion of the membrane at all frequencies, which is equivalent to raising the effective mechanical temperature. In this way, the root mean square (rms) displacement is increased, thereby facilitating the detection of mechanical modes. The noise signal has a bandwidth of 1 MHz, larger than the spectral width of the mechanical modes, and an amplitude *V*_M_ = 2.7 V_rms_. Figure 2(a) shows the mechanical sidebands at *f*_C_ + *f*_*i*,*j*_. To distinguish mechanical sidebands from other parasitic signals, we increase *P*_C_ until we observe a frequency shift (See Supplemental Material for further details). The fundamental mode of the membrane is at *f*_0_ = 78.573 ± 0.002 kHz. We fit each mechanical sideband with a Lorentzian (Fig. 2(b–d)). As in Fig. 1(d), we observe double peaked sidebands (Fig. 2(c,d)).

The broad cavity bandwidth allowed us to measure the actuated membrane’s motion in real time. We excite the membrane with white noise whilst driving the cavity at *f*_C_. In order to record the motion in real time, the cavity output signal is mixed with a local oscillator. The output signal (Fig. 2(e)) shows clear sinusoidal oscillations evidencing the membrane’s motion.

**Figure 3:**
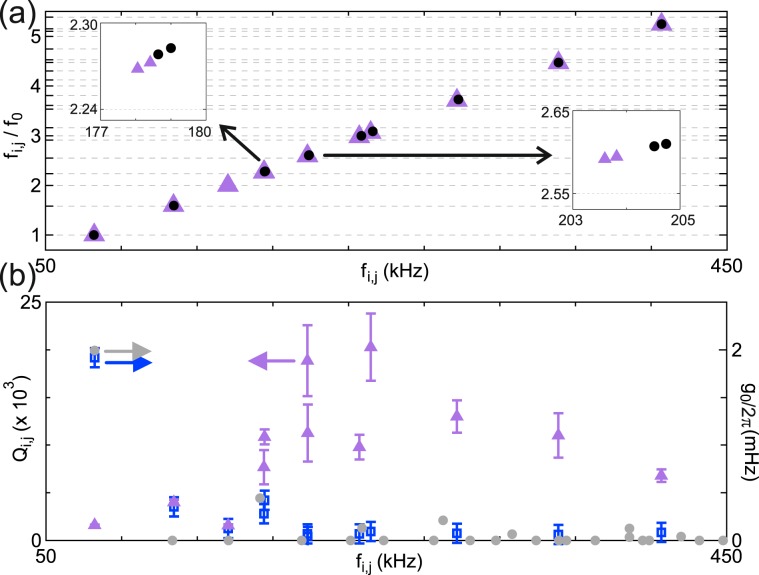
(**a**) Frequency ratios of the mechanical resonances extracted from Fig. 1(d) (circles) and Fig. 2(a) (triangles). Grid lines indicate theoretical ratios. Errors in frequency and frequency ratios are smaller than symbols. Insets: zoom-in showing selected nearly degenerate modes. (**b**) Triangles: Quality factors extracted from Lorentzian fits as in Fig. 2 for each mechanical mode. Error bars were obtained by combining fit results for different values of *V*_M_. Squares: Single-photon coupling strength calculated for each mechanical mode. Circles: Single-photon coupling strength estimated from a parallel capacitor model, corrected for different mode profiles, for *f*_*i*,*j*_ obtained from theoretical ratios.

We plot *f*_*i*,*j*_/*f*_0_ as a function of *f*_*i*,*j*_ in Fig. 3(a) for all mechanical resonances observed in Figs. 1(d) and 2(a). Lower frequency modes show better agreement with theoretical ratios than higher frequency modes. From the Lorentzian fits of each mechanical sideband, we extract the mechanical quality factors *Q*_i,j_ and single photon coupling strengths *g*_0_ (Fig. 3(b)). These values of *Q*_i,j_, measured in the spectral response, are sensitive both to dissipation and dephasing^20^. The fundamental mode has a quality factor *Q*_0_ = (1.6 ± 0.1) × 10^3^ and the highest quality factor measured was (20 ± 4) × 10^3^ for the mode at 241 kHz. Different modes and even nearly degenerate mechanical modes have significant differences in their values of *Q*_i,j_, as previously reported^21,22^. The values of *Q*_i,j_ vary slightly as a function of *V*_M_ ranging from 0.1 to 3.6 V_rms_, but they do not show a specific trend (See Supplemental Material for data on the mechanical quality factors as a function of V_M_). The error bars correspond to this deviation in the values of *Q*_i,j_.

We extract *g*_0_ for each mechanical mode from the effective thermomechanical power, i.e. from the area of the corresponding sideband in Fig. 3(b) (See Supplemental Material for details of the estimation of the single-photon coupling from noise measurements). For the first mechanical mode, we obtain *g*_0_/2*π* = 1.9 ± 0.1 mHz. The cavity drive was 5 dBm at port 1, yielding a multiphoton coupling strength $g/2\pi =\sqrt{{n}_{{\rm{C}}}}{g}_{0}/2\pi  \sim 4.4$ kHz, where *n*_C_ is the mean cavity photon number. As expected, the first mode couples more strongly than higher frequency modes, due to its mode profile and larger zero point motion (See Supplemental Material for details).

These values of *g*_0_ can be compared with the predictions of Eq. 1. Taking *C*_T_ and *d* from the circuit model, and using that $\frac{\partial {C}_{{\rm{C}}}}{\partial u} \sim \frac{{\varepsilon }_{0}a}{{d}^{2}}$ for a parallel-plate capacitor (with a known prefactor to account for the mechanical mode profile of the membrane), gives a coupling strength *g*_0_/2*π* ≈ 2 mHz for the fundamental mode^18^. The estimated values of *g*_0_ are similar to the ones extracted from the sideband powers.

We can estimate the vibrational amplitude sensitivity *S*_*u*_ given an amplifier voltage sensitivity ${S}_{v}=\sqrt{{k}_{{\rm{B}}}{T}_{{\rm{N}}}{Z}_{0}},$ where *k*_B_ is the Boltzmann constant, *Z*_0_ ≡ 50 Ω and *T*_N_ ~ 293 K the system noise temperature. We can write ${S}_{u}={S}_{v}/(\frac{\partial |{S}_{21}|}{\partial {C}_{{\rm{T}}}}\frac{\partial {C}_{{\rm{C}}}}{\partial u}{V}_{{\rm{in}}})$ where *V*_in_ is the voltage at electrode 1 corresponding to *P*_C_. Taking $\frac{\partial |{S}_{21}|}{\partial {C}_{{\rm{T}}}}$ from the circuit model and $\frac{\partial {C}_{{\rm{C}}}}{\partial u} \sim 1.7\times {10}^{-7}$ F/m extracted from the sideband’s area corresponding to the fundamental mode, we obtain ${S}_{u}=0.4\,{\rm{pm}}/\sqrt{{\rm{Hz}}}$. This value is comparable to that obtained for a suspended carbon nanotube device at cryogenic temperatures^12^.

Finally, we measure optomechanically induced transparency (OMIT) and absorption (OMIA), which are signatures of optomechanical coupling and demonstrate that the membrane’s motion can be actuated by radiation-pressure alone. OMIT and OMIA are characterized by the emergence of a transparency or absorption window in |S_21_| when a strong drive tone (*f*_D_) and a weak probe tone (*f*_P_) are injected into the cavity, and the frequency difference between these tones *δf* = *f*_P_ − *f*_D_ coincides with the frequency of a mechanical mode^15^. When this condition is met, the beat between the drive and the probe field excites the membrane’s motion and the destructive (constructive) interference of excitation pathways for the intracavity probe field results in a transparency (absorption) window in |S_21_|.

**Figure 4:**
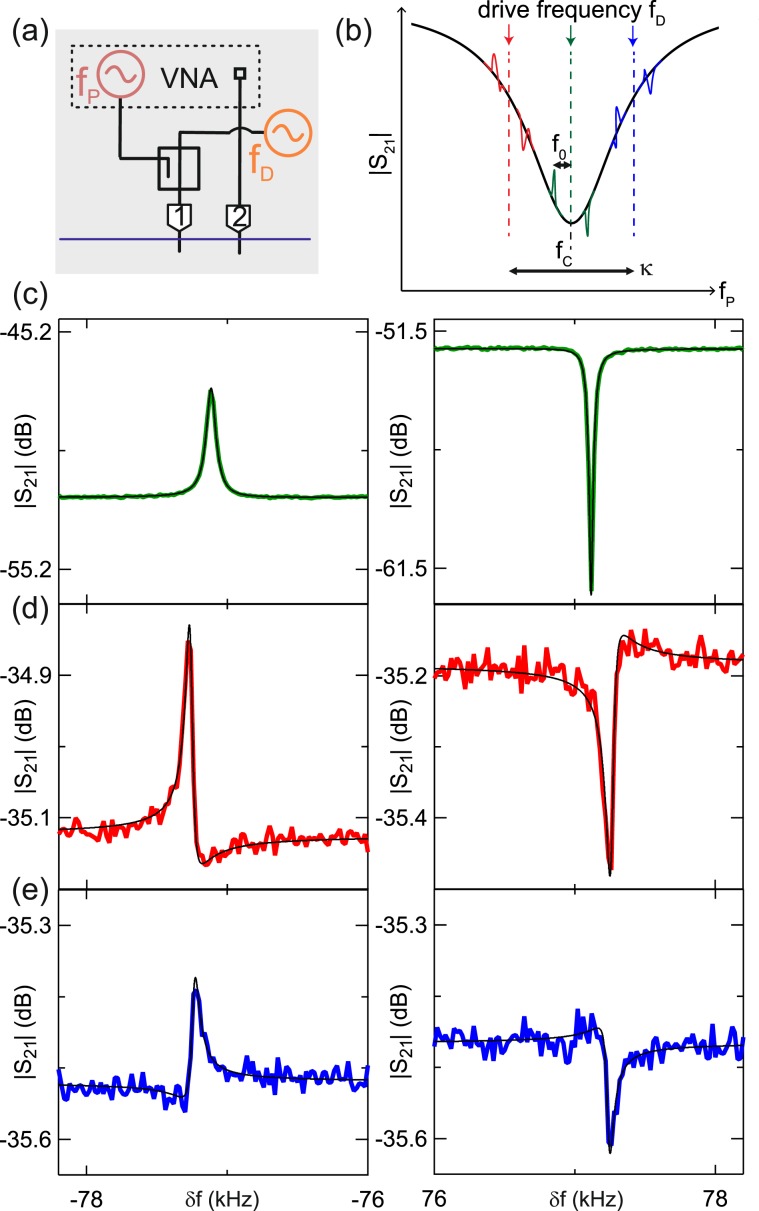
(**a**) Schematic of signal injection and detection for OMIT/OMIA measurements. A tone with frequency *f*_D_ and a tone with frequency *f*_P_ are injected through a directional coupler via port 1. We monitor |S_21_| with a vector network analyzer. (**b**) Schematic showing each of the drive frequencies injected and a cartoon of the mechanical sidebands appearing at *f*_D_ ± *f*_0_. Drive frequencies were *f*_D_ ~ *f*_C_ (green), *f*_D_ ~ *f*_C_ − *κ*/2 (red) and *f*_D_ ~ *f*_C_ + *κ*/2 (blue). The corresponding mechanical sidebands are shown in panels (**c**–**e**) respectively. (**c**–**e**) Measured |S_21_| as a function of *δf* showing mechanical sidebands at *δf* ~ −*f*_0_ (left panel) and *δf* ~ *f*_0_ (right panel) for each of the values of *f*_D_ considered in (**b**). At port 1, the drive power was 5 dBm and the probe power −27.5 dBm. Black curves are a fit to the data.

To show optomechanical control, we measured OMIT and OMIA by injecting a strong tone at frequency *f*_D_ and a weak tone at frequency *f*_P_ through a directional coupler at port 1 (Fig. 4(a)). We injected three different drive frequencies (Fig. 4(b)); *f*_D_ ~ *f*_C_ − *κ*/2 (red detuned), *f*_D_ ~ *f*_C_ (resonant) and *f*_D_ ~ *f*_C_ + *κ*/2 (blue detuned). When *f*_D_ ~ *f*_C_, a peak (dip) is observed at *f*_D_ − (+)*f*_0_ (Fig. 4(c)). When *f*_D_ ~ *f*_C_ ± *κ*/2, we observe Fano-like features at *f*_D_ ± *f*_0_ (Fig. 4(d,e)). We do not expect complete extinction or revival of the reflected signal as the system is well within the unresolved sideband limit.

We fitted OMIT and OMIA features by modelling the transmission of the probe field (See Supplemental Material for details of the estimation of the single-photon coupling from OMIT measurements). From the fit of the spectral features, we extract *f*_0_ = 77.2 ± 0.1 kHz, *Q*_0_ = (1.2 ± 0.1)× 10^3^ and *g*_0_/2*π* = 2.3 ± 0.3 mHz. Error bars were obtained by combining fit results of the six curves in Fig. 4(c–e). The values obtained for *f*_0_ and *Q*_0_ are similar to those extracted from the Lorentzian fits as in Fig. 2(b). The value of *g*_0_ is in agreement with that extracted from Fig. 2(a) and estimated from the parallel plate capacitor approximation.

## Discussion

To conclude, we have characterized several modes of a silicon nitride membrane with an off-resonant rf circuit at room temperature, deeper in the unresolved sideband regime than previously explored. Our cavity allows for the injection of noise to actuate the motion of the membrane and effectively increase its mechanical mode temperature. We achieve a sensitivity of $0.4\,{\rm{pm}}/\sqrt{{\rm{Hz}}}$, a tenfold improvement to that reported in ref. ^14^ although the smaller size of their mechanical resonator makes direct comparison difficult. Our results show that our on-chip platform can be used for membrane actuation and characterization. The readout circuit operates at a convenient frequency and does not require the cavity to be tuned into resonance with the membrane as in other approaches. It therefore has applications in mechanical sensing and microwave-to-optical conversion. Thanks to the large bandwidth of the cavity we have also measured the actuated membrane’s motion in real time. Finally we have observed OMIT and OMIA, from which we obtained a separate measure of the frequency, quality factor and coupling strength of the fundamental mode.

## Data Availability

The data analysed during the current study are available from the corresponding author on reasonable request.

## Supporting Information

Supporting Information.
