# Supplementary material for: Radio-frequency optomechanical characterization of a silicon nitride drum
Source: Sci Rep. 2020 Feb 3;10:1654. doi: 10.1038/s41598-020-58554-x (PMC6997228; doi:10.1038/s41598-020-58554-x)
Supplement: Supplementary file 1 — Supporting Information. [file 41598_2020_58554_MOESM1_ESM.pdf]

# Radio-frequency optomechanical characterization of a silicon nitride drum - Supplementary Information

**A. N. Pearson[1], K. E. Khosla[2,3], M. Mergenthaler[1], G.A.D. Briggs[1], E.A. Laird[4], and N. Ares[1,*]**

[1]Department of Materials, University of Oxford, Parks Road, Oxford OX1 3PH, United Kingdom
[2]Center for Engineered Quantum Systems and The School of Mathematics and Physics, The University of Queensland, Brisbane, Queensland 4067, Australia
[3]QOLS, Blackett Laboratory, Imperial College London, London SW7 2AZ, United Kingdom
[4]Department of Physics, Lancaster University, Lancaster, LA1 4YB, United Kingdom
[*]natalia.ares@materials.ox.ac.uk

## 1 Cavity characterisation

### 1.1 Fit of the cavity transmission

The response of the cavity, Fig. 1 (b) of the main text, was characterized by fitting the measured transmission to the following expression:

$$S_{21}(f_{\rm P}) = \alpha e^{i\phi} + (1-\alpha)(1 - \frac{\kappa_e}{-2\pi i(f_{\rm P} - f_{\rm C}) + \kappa/2}) \tag{1}$$

where $\alpha$ is the isolation of the directional coupler and $\phi$ a phase[1]. The cavity resonance frequency is $f_{\rm C}$ , $\kappa_e$ is the external dissipation rate and $\kappa$ the total dissipation rate of the cavity.

From a fit with a fixed value of $\alpha = 0.001$ from the directional coupler data sheet and correcting for an insertion loss of -16.4 dB, we extract $\phi = (0.9 \pm 0.1)\pi$ rad, $f_{\rm C} = 209.23 \pm 0.01$ MHz, $\kappa/(2\pi) = 28.20 \pm 0.01$ MHz and $\kappa_e/(2\pi) = 15.80 \pm 0.01$ MHz.

### 1.2 Circuit simulation

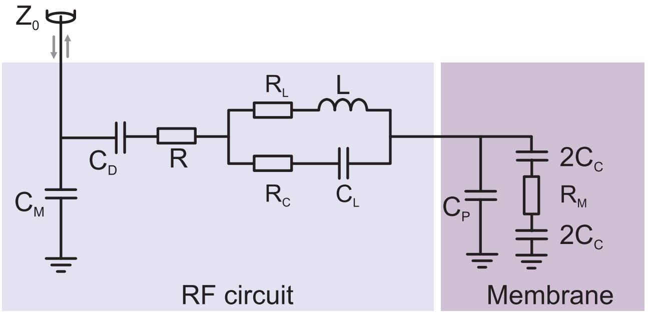


**Figure 1.** Circuit model. Capacitors $C_{\rm M}$ and $C_{\rm D}$ are taken as simple lumped elements, including any parasitic capacitances in parallel. Elements $R_{\rm L}$, $R_{\rm C}$ and $C_{\rm L}$ model parasitic contributions to the impedance of the inductor $L$. The effective resistance $R$ models other losses in the circuit. The membrane is modelled by the combination of $R_{\rm M}$ and $C_{\rm C}$, the capacitance between the metallised area of the membrane and both antenna electrodes. To each electrode, the membrane-electrode capacitance is $2C_{\rm C}$, which when summed together in series gives $C_{\rm C}$.

To extract circuit parameters the cavity response can also be fit using the circuit model in Fig. 1, as shown in Fig. 1 (b). The capacitors $C_{\rm D}$ and $C_{\rm M}$ are taken as simple lumped elements. We consider the capacitor formed between the electrodes and the metalized membrane, $C_{\rm C}$, formed by two electrode-membrane capacitors of value $2C_{\rm C}$, as well as the capacitance between antenna electrodes, $C_{\rm A}$. The inductor is modeled as a network of elements as shown, which simulate its self-resonances and losses. The membrane has a resistance $R_M$ and other losses in the circuit are modeled by an effective resistance $R$.

The reflection coefficient $\Gamma$ is then equal to

$$\Gamma(f_P) = \frac{Z_{tot}(f_P) - Z_0}{Z_{tot}(f_P) + Z_0}, \tag{2}$$

where $Z_{tot}$ is the total impedance from the circuit's input port and $Z_0 = 50\ \Omega$ is the line impedance. We relate the measured transmission $S_{21}$ to $\Gamma$ by assuming a constant overall insertion loss $A$, incorporating attenuation in the lines, the coupling of the directional coupler, and the gain of the amplifier, such that

$$|S_{21}(f_P)| = A|\Gamma(f_P)|. \tag{3}$$

Fitting to Eq. (1), we take $C_D = 10$ pF from the known component value, and $L = 223$ nH, $R_L = 3.15 \times 10^{-4}\ \Omega \times \sqrt{f_P\ [\text{Hz}]}$, $R_C = 25\ \Omega$ and $C_L = 0.082$ pF from the datasheet of the inductor. For the resistance of the aluminium film, we estimate $R_M$ = 7.5 $\Omega$ using the resistivity of aluminium (15 $\mu\Omega$ cm) and the known film thickness (20 nm)[2]. We have estimated 1.4 pF from a COMSOL model of the antenna electrodes and we have added a parasitic capacitance of 0.7 pF based on previous work[3] making a total parasitic capacitance $C_P$ of 2.1 pF. Fit parameters are then $A$, $C_M$, $R$, and $C_C$. From the fit we obtain $A = -16.657 \pm 0.001$ dB, $C_M = 20.93 \pm 0.01$ pF, $R = 15.87 \pm 0.01\ \Omega$ and $C_C = 1.6432 \pm 0.0001$ pF.

The cavity coupling to the membrane displacement is given by $\frac{df_C}{du} = \frac{\partial f_C}{\partial C_T}\frac{\partial C_C}{\partial u}$ and $\frac{\partial f_C}{\partial C_T} \approx 1/(4\pi\sqrt{LC_T^3})$ where $C_T \equiv C_P + C_A + C_C$. Although this approximation applies strictly only for a simple LC resonator, we confirmed numerically that this procedure gives a good approximation for $\frac{\partial f_C}{\partial C_T}$.

# 2 Mechanical Characterization

## 2.1 Mechanical quality factors

From Lorentzian fits as in Fig. 2(b-d) of the main text we can extract $Q_{i,j}$. We extracted $Q_{i,j}$ for different values of $V_M$ (Fig. 2). At low $V_M$, the mechanical sidebands became fainter, and some modes could not be reliably fitted. The values of $Q_{i,j}$ do not show a trend as a function of $V_M$. The error bars in $Q_{i,j}$ in Fig. 3(b) of the main text were obtained, for each mechanical mode, by combining $Q_{i,j}$ for different values of $V_M$.

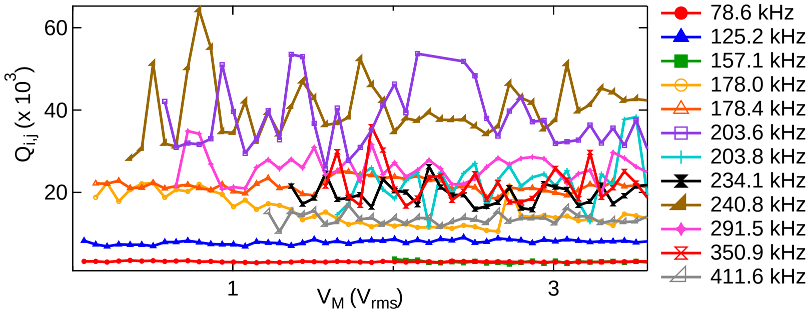


**Figure 2.** Extracted $Q_{i,j}$ as a function of $V_M$ for each mechanical mode observed.

## 2.2 Mechanical sidebands as a function of cavity drive

In order to distinguish the mechanical sidebands in Fig. 2(a) from parasitic resonances we measure them as a function of $P_C$ (Fig. 3), as the frequency of the mechanical modes decreases for the highest values of $P_C$. This might have to do with heating of the membrane surface and thereby a decrease in its tension. From the circuit model we can calculate the power dissipated in the membrane. For $P_C = 15$ dBm, the power dissipated is $\sim 4\ \mu$W. We estimate the maximum temperature increase by assuming that all the dissipated power is emitted as thermal radiation. Taking the emissivity of aluminum as 0.09 and applying the Stefan-Boltzmann law leads to an estimated temperature increase of 4.5 K. For $P_C = 20$ dBm, the temperature difference is $\sim 13.5$ K. A similar calculation confirms that the injected noise does not change the temperature of the membrane significantly.

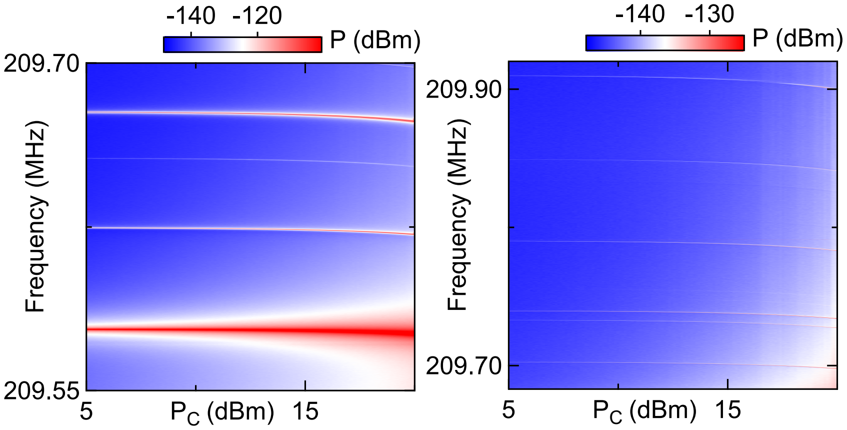


**Figure 3.** Power spectrum as a function of frequency and $P_C$ for $V_M = 2.7$ V$_{rms}$. The cavity drive is at 209.5 MHz. Several mechanical sidebands can be observed. The first few mechanical modes are displayed in (a) and the higher frequency mechanical modes are displayed in (b). As $P_C$ increases, the mechanical sidebands are brighter and their frequency shifts to lower values for $P_C \gtrsim 15$ dBm.

## 3 Electromechanical coupling

### 3.1 Extraction of $g_0$ from mechanical sidebands

From the area below the sidebands in Fig. 2(a) of the main text, we extracted the values of $g_0$ plotted in Fig. 3(b) of the main text. In this Section we will derive the expression relating $g_0$ to the effective thermomechanical power ($P_{side}$) extracted from this area. We start with $g_0/2\pi = \frac{df_C}{du} u_{ZP} = \frac{\partial f_C}{\partial C_T}\frac{\partial C_C}{\partial u} u_{ZP}$. As discussed in Section 1.2 $\frac{\partial f_C}{\partial C_T} \approx (4\pi\sqrt{LC_T^3})^{-1}$ giving

$$g_0/2\pi \approx (4\pi\sqrt{LC_T^3})^{-1}\frac{\partial C_C}{\partial u}u_{ZP}, \tag{4}$$

where $u_{ZP} = \sqrt{\hbar/(4\pi m f_{i,j})}$ is the zero point motion of the membrane with effective mass $m \sim 4.5 \times 10^{-10}$ kg. We choose to normalize the mode eigenfunctions such that the effective mass equals the mass of the suspended segment for all modes[4]. The value of $L$ is known, $C_T$ is obtained from the circuit model fit and $u_{ZP}$ can be estimated from the values of $f_{i,j}$ extracted from the Lorentzian fit of the mechanical sidebands. To estimate $\frac{\partial C_C}{\partial u}$, we write $P_{side}$, which obeys

$$P_{side} = \tilde{P}_C\, 2\bar{n}_{i,j}\left(\frac{\kappa_e}{\kappa}\right)^2\frac{g_0^2}{(\frac{\kappa}{2})^2+(2\pi f_{i,j})^2}, \tag{5}$$

where $\tilde{P}_C$ is the cavity drive $P_C$ having taken into account the overall insertion loss and $\bar{n}_{i,j}$ is the phonon occupancy of the mode[5]. Replacing $g_0^2$ with Eq. (4),

$$P_{side} \approx \tilde{P}_C\, 2\bar{n}_{i,j}\left(\frac{\kappa_e}{\kappa}\right)^2\frac{\left(\frac{\partial C_C}{\partial u}\right)^2 u_{ZP}^2}{((\frac{\kappa}{2})^2+(2\pi f_{i,j})^2)(2\sqrt{LC_T^3})^2}. \tag{6}$$

For the measurements in Fig. 2 of the main text $P_C = 5$ dBm at port 1. The values of $\kappa$, $\kappa_e$ and $A$ are obtained from the cavity characterization (Section 1.1) and the circuit model fit (Section 1.2).

We now write $\bar{n}_{i,j}$,

$$\bar{n}_{i,j} = \frac{m\,(2\pi f_{i,j})^2\langle\delta u_{i,j}^2\rangle}{\hbar\,(2\pi f_{i,j})}, \tag{7}$$

where $\delta u_{\text{i,j}}$ is the membrane displacement from its equilibrium position. In order to estimate the rms value $\langle \delta u_{\text{i,j}}^2 \rangle$, we write the effective electromechanical force on the membrane,

$$F(t) = \frac{1}{2} V^2(t) \frac{\partial C_C}{\partial u}, \tag{8}$$

where $V(t) = V_{\text{DC}} + \delta V(t)$. The time-independent part $V_{\text{DC}} = 15$ V is much larger than the time-dependent part $\delta V(t)$. The time-dependent part of $F(t)$ is to lowest order

$$\delta F(t) = V_{\text{DC}} \frac{\partial C_C}{\partial u} \delta V(t), \tag{9}$$

where we assumed this electronic fluctuating force is much larger than the thermal noise.

In the frequency domain, the displacement is

$$\delta u_{\text{i,j}}(f) = \chi_{\text{i,j}}(f) \delta F(f), \tag{10}$$

where the mechanical susceptibility is $\chi_{\text{i,j}}(f) = \frac{1}{4\pi^2 m} [f_{\text{i,j}}^2 - f^2 + i f f_{\text{i,j}}/Q_{\text{i,j}}]^{-1}$[6,7]. The values of $Q_{\text{i,j}}$ can be extracted from the Lorentzian fit of the mechanical sidebands (Section 2.1). We calculate $[\delta u_{\text{i,j}}^2]_{\text{rms}}$,

$$[\delta u_{\text{i,j}}^2]_{\text{rms}} = V_{\text{DC}}^2 \left( \frac{\partial C_{\text{C}}}{\partial u} \right)^2 \int_{-\infty}^{\infty} \int_{-\infty}^{\infty} df df' \langle \delta V(f) \delta V(f') \rangle \langle \chi_{\text{i,j}}(f) \chi_{\text{i,j}}(f') \rangle, \tag{11}$$

where $\chi_{\text{i,j}}(f)$ and $\delta V(f)$ are uncorrelated. The fluctuating voltage $\delta V(f)$ is assumed to be well approximated by white noise over the frequency range of interest, with $\langle \delta V(f) \delta V(f') \rangle = \frac{S_V^2}{2} \delta(f + f')$, where $S_V$ is the single-sided white noise power spectrum of the driving voltage $V$. We obtain

$$[\delta u_{\text{i,j}}^2]_{\text{rms}} = V_{\text{DC}}^2 \left( \frac{\partial C_{\text{C}}}{\partial u} \right)^2 \left( \int_0^{\infty} df |\chi_{\text{i,j}}(f)|^2 \right) \frac{S_V^2}{2}, \tag{12}$$

given that $\chi_{\text{i,j}}(f)^* = \chi_{\text{i,j}}(-f)$.

We can now rewrite Eq. 7,

$$\bar{n}_{\text{i,j}} = m(2\pi f_{\text{i,j}}/\hbar)\, V_{\text{DC}}^2 \left( \frac{\partial C_{\text{C}}}{\partial u} \right)^2 \frac{S_V^2}{2} \int df |\chi_{\text{i,j}}(f)|^2. \tag{13}$$

Replacing Eq. 13 in Eq. 5, we obtain the following expression,

$$\frac{\partial C_{\text{C}}}{\partial u} = \sqrt[4]{\frac{P_{\text{side}}}{\tilde{P}_{\text{C}}} \left( \frac{\kappa}{\kappa_e} \right)^2 \frac{((\frac{\kappa}{2})^2 + (2\pi f_{\text{i,j}})^2)(2LC_{\text{T}}^3)}{V_{\text{DC}}^2 \frac{S_V^2}{2} \int_0^{\infty} df |\chi_{i,j}(f)|^2}}. \tag{14}$$

With the amplitude of the driving noise set to $V_{\text{M}} = 2.7\ \text{V}_{\text{rms}}$, the corresponding spectral density is measured as $S_V^2 = (3 \pm 0.1) \times 10^{-10}$ V$^2$/Hz and we calculate $\int_0^{\infty} df |\chi_{\text{i,j}}(f)|^2$ numerically. Once we estimate $\frac{\partial C_{\text{C}}}{\partial u}$, Eq. 4 gives us $g_0$ for each observed mechanical mode (Fig. 3 of the main text). The error in this quantity reflects uncertainty in the cavity characterization ($A$, $\kappa$ and $\kappa_e$), the circuit model fit ($C_{\text{T}}$), the measurement of $S_V^2$, $P_{\text{side}}$ and the fit of the mechanical sidebands ($f_{\text{i,j}}$ and $Q_{\text{i,j}}$).

We also calculate the rms displacement $[\delta u_{\text{i,j}}]_{\text{rms}}$ corresponding to the mechanical sidebands in Fig. 3 of the main text using Eq. 12. For the fundamental mode, $[\delta u_0]_{\text{rms}} \sim 14$ nm (Fig. 4). For comparison, for a thermal state at 293 K $[\delta u_0]_{\text{rms}} = u_{\text{ZP}} \sqrt{k_{\text{B}} T / h f_0} \sim 4$ pm. Therefore the white noise power spectrum of the electronic force is much larger than the power spectrum of the thermal noise $V_{\text{DC}}^2 S_V^2 (\partial C_{\text{C}}/\partial u)^2 \gg 4\pi k_{\text{B}} T f_{\text{i,j}}/Q_{\text{i,j}}$, justifying the approximation in Eq. 9.

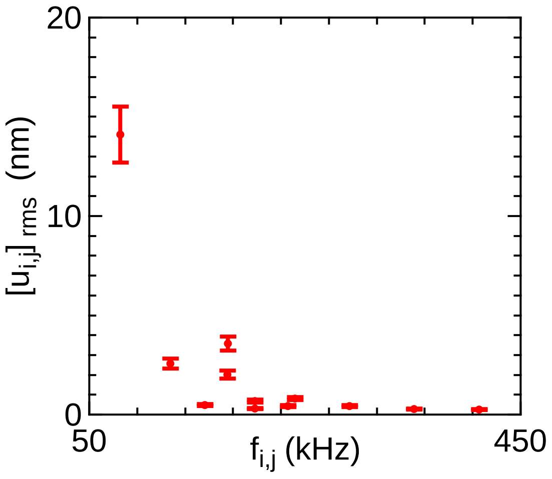


**Figure 4.** Extracted $[\delta u_{\rm i,j}]_{\rm rms}$ as a function of $f_{\rm i,j}$ extracted from the measurements shown in Fig. 2 of the main text.

### 3.2 Mode profile correction to $g_0$

The mode profile modifies $\frac{\partial C_{\rm C}}{\partial u}$ and thus $g_0$. For modes with $i \times j$ even, the sections of the membrane moving away from the antenna and the sections moving towards the antenna are equal, and therefore the net $\frac{\partial C_{\rm C}}{\partial u}$ is close to zero, and $g_0 \sim 0$. For $i \times j$ odd, there is always a section of the membrane which does not have a counterpart moving out of phase. Therefore, for odd mode profiles $\frac{\partial C_{\rm C}}{\partial u}$, and thus $g_0$, are reduced by a factor $1/(i \times j)$.

## 4 Electromechanically induced transparency

The transmission of our circuit in the presence of a weak probe tone at $f_{\rm P}$ and a strong drive tone at $f_{\rm D}$, as shown in the Fig.4(c-e) of the main text, is [6,8]:

$$|S_{21}(\delta f)| = 1 - \frac{(1 - i\mathscr{F}(\delta f))\kappa_e}{2\pi i(\Delta + \delta f) + \kappa/2 + 4\pi\Delta F(\delta f)}, \quad (15)$$

where $\delta f = f_{\rm P} - f_{\rm D}$, $\Delta = f_{\rm D} - f_{\rm C}$ and

$$\mathscr{F}(\delta f) = \frac{\hbar\, g_0^2\, \chi_0(\delta f)\, \kappa_e\, S_{\rm in}^2}{u_{\rm ZP}^2(2\pi i(\delta f - \Delta) + \kappa/2)((2\pi\Delta)^2 + (\kappa/2)^2)}. \quad (16)$$

We define $\chi_0$ as the mechanical susceptibility of the fundamental mode and $S_{\rm in}$ as the photon flux incident from the drive tone,

$$S_{\rm in}^2 = \frac{\tilde{P}_{\rm D}}{2\pi\hbar f_{\rm D}}, \quad (17)$$

where $\tilde{P}_{\rm D}$ is the power of the drive tone ($P_{\rm D}$) having taken into account the overall insertion loss. For the measurements in Fig. 4 of the main text $P_{\rm D} = 5$ dBm.

Using the values of $\kappa/(2\pi)$ and $\kappa_e/(2\pi)$ extracted in section 1.1, we fitted the curves in each panel of Fig. 4(c-e) of the main text with equation 15. In this way, we obtained the reported values for $Q_0$, $f_0$ and $g_0/2\pi$. The uncertainties in these quantities reflect the variance among the values obtained for each curve.

To estimate the amplitude of the membrane's motion we consider parametric amplification of the oscillator due to the beat frequency between the drive and probe tones[8]. For the OMIT measurement, the probe is 32.5 dB weaker than the drive (see Fig. 4 of the main text), hence the intracavity photon number is modulated by $N \approx 2 \times 10^{-32.5/20} n_{\rm C}$. Here we have neglected any difference due to the cavity resonance as both tones are well within the linewidth. The circulating photon number is

estimated at $n_C \approx 5.2 \times 10^{12}$ (at 5 dBm). For OMIT the beat frequency is at $f_0$, hence the oscillator sees the parametric force $F = \hbar g_0 N \cos(2\pi f_0 t)/u_{ZP}$. This force results in a coherent amplitude $u_{max} = \frac{\hbar g_0 Q_0 N}{m u_{ZP}(2\pi f_0)^2} \sim 8.6$ nm. This is significantly above the thermal rms motion.

## References


**1.** Singh, V. *et al.* Optomechanical coupling between a multilayer graphene mechanical resonator and a superconducting microwave cavity. *Nat. Nanotechnol.* **9**, 820–824, DOI: 10.1038/nnano.2014.168 (2014).

**2.** Lacy, F. Developing a theoretical relationship between electrical resistivity, temperature, and film thickness for conductors. *Nanoscale Res. Lett.* **6**, 1–26, DOI: 10.1186/1556-276X-6-636 (2011).

**3.** Ares, N. *et al.* Sensitive radio-frequency measurements of a quantum dot by tuning to perfect impedance matching. *Phys. Rev. Appl.* **5**, 34011, DOI: 10.1103/PhysRevApplied.5.034011 (2016).

**4.** Poot, M. & van der Zant, H. S. J. Mechanical systems in the quantum regime. *Phys. Rep.* **511**, 273–335, DOI: 10.1016/j.physrep.2011.12.004 (2012).

**5.** Yuan, M., Singh, V., Blanter, Y. M. & Steele, G. A. Large cooperativity and microkelvin cooling with a three-dimensional optomechanical cavity. *Nat. Commun.* **6**, 8491, DOI: 10.1038/ncomms9491 (2015).

**6.** Aspelmeyer, M., Kippenberg, T. J. & Marquard, F. Cavity optomechanics. *Rev. Mod. Phys.* **86**, 1391–1452, DOI: 10.1103/RevModPhys.86.1391 (2014).

**7.** Lehnert, K. W. Introduction to Microwave Cavity Optomechanics BT - Cavity Optomechanics: Nano- and Micromechanical Resonators Interacting with Light. 233–252, DOI: 10.1007/978-3-642-55312-7_11 (Springer Berlin Heidelberg, Berlin, Heidelberg, 2014).

**8.** Weis, S. *et al.* Optomechanically induced transparency. *Science* **330**, 1520–3, DOI: 10.1126/science.1195596 (2010).
